# Determinants of patient satisfaction with outpatient health services at public and private hospitals in Addis Ababa, Ethiopia

**DOI:** 10.4102/phcfm.v4i1.384

**Published:** 2012-08-24

**Authors:** Tayue Tateke, Mirkuzie Woldie, Shimeles Ololo

**Affiliations:** 1 College of Medicine and Health Sciences, Arbaminch University, Ethiopia; 2 Department of Health Services Management, Jimma University, Ethiopia

## Abstract

**Background:**

Patients have explicit desires or requests for services when they visit hospitals. However, inadequate discovery of their needs may result in patient dissatisfaction. This study aimed to determine the levels and determinants of patient satisfaction with outpatient health services provided at public and private hospitals in Addis Ababa, Central Ethiopia.

**Methods:**

A comparative cross-sectional study was conducted from 27 March to 30 April 2010. The study included 5 private and 5 public hospitals. Participants were selected using systematic random sampling. A pre-tested and contextually prepared structured questionnaire was used to conduct interviews. Descriptive statistics, analysis of variance, factor analysis and multiple linear regressions were performed using computer software (SPSS 16.0).

**Results:**

About 18.0% of the patients at the public hospitals were very satisfied whilst 47.9% were just satisfied with the corresponding proportions a bit higher at private hospitals. Self-judged health status, expectation about the services, perceived adequacy of consultation duration, perceived providers’ technical competency, perceived welcoming approach and perceived body signalling were determinants of satisfaction at both public and private hospitals.

**Conclusions:**

Although patients at the private hospitals were more satisfied than those at the public hospitals with the health care they received, five of the predictors of patient satisfaction in this study were common to both settings. Thus, hospitals in both categories should work to improve the competencies of their employees, particularly health professionals, to win the interests of the clients and have a physical structure that better fits the expectations of the patients.

## Abstrait

**Les facteurs de satisfaction des patients quant aux soins ambulatoires dans les hôpitaux publics et privés à Addis Abeba, Éthiopie**

**Contexte:**

Les patients ont des souhaits et des demandes explicites concernant les services lors des visites à l'hôpital. Cependant, une réponse inappropriée à leurs besoins peut entraîner l'insatisfaction du patient. Cette étude a pour but de déterminer les niveaux et les facteurs de satisfaction du patient quant aux soins ambulatoires fournis dans les hôpitaux publics et privés à Addis Abeba, en Éthiopie centrale.

**Méthodes:**

Une étude transversale comparative a été réalisée du 27 mars au 30 avril 2010. L’étude concernait 5 hôpitaux publics et 5 hôpitaux privés. Les participants ont été sélectionnés en utilisant la technique systématique d’échantillonnage aléatoire. Un questionnaire pré-testé et mis en contexte a été utilisé pour mener à bien les entretiens. Les statistiques descriptives, l'analyse de la variance, l'analyse des facteurs et la régression linéaire multiple sont effectuées au moyen d'un logiciel informatique (SPSS 16.0).

**Résultats:**

Près de 18% des patients des hôpitaux publics étaient très satisfaits alors que 47.9% étaient seulement satisfaits, les proportions étant légèrement supérieures dans les hôpitaux privés. L’état de santé auto-déclaré, les attentes concernant les services, la perception de la pertinence de la durée des consultations, la perception de la compétence technique des fournisseurs de soins, la perception de l'accueil et la perception du langage corporel étaient déterminants pour la satisfaction, dans les hôpitaux privés comme publics.

**Conclusions:**

Bien que les patients des hôpitaux privés soient plus satisfaits que ceux des hôpitaux publics quant aux soins de santé qu'ils ont reçus, cinq des indicateurs de satisfaction des patients de cette étude étaient communs aux deux contextes. Ainsi, les hôpitaux des deux catégories devraient faire un effort afin d'améliorer la compétence de leurs employés, en particulier les professionnels de la santé, de concilier les intérêts de leurs clients et de disposer d'une infrastructure physique mieux adaptée aux attentes des patients.

## Introduction

### Key focus

Though each group has its own specific and different interests and opinions, the definition, measurement and improvement of health care quality have been a primary issue for health care providers, health service managers and those who commission the service for patients for centuries.^1^ However, in both developing and developed countries, there has been an implicit acknowledgment that many health services do not meet minimum standards for clinical effectiveness or client satisfaction.^2^

#### Background

In a study conducted amongst seven developing countries, researchers who directly observed the clinical practice, found that 75% of cases were not adequately diagnosed, treated or monitored.^3^ Though the most frequent explanation for the variation and low-quality care in the developing world was lack of resources. One study noted that despite having high expenditure and adequate facilities, patients were often not satisfied with the health care they received.^4^

Patients have explicit desires or requests for services when they visit hospitals. However, many cases of patient dissatisfaction can occur due to inadequate discovery of their needs.^3^ According to Donabedian, patient satisfaction should be investigated since it is an objective of care, a consequence of that care (outcome) that can contribute to the effects of care, as a satisfied patient is more likely to comply with advice, and it is the patient's judgment on the care that has been provided.^5^

Moreover, there is growing consensus that assessment of the quality of hospital services should be based in part, on patients’ perceptions of overall care and patient satisfaction.^6^ The dominance of market-oriented approaches to reforms in health care delivery and cost, and the emergence of a normative perspective on clinical practice that emphasises the need to deliver patient-centred care, are also driving attention to patient perceptions of quality.^7, 8, 9^

Different studies have pointed out that the level of satisfaction in different types of health facilities and hospitals vary. Studies in Ethiopia have reported overall satisfaction levels ranging from 52% to 57%.^10, 11, 12^ Studies in Bangladesh showed that greater levels of satisfaction were observed in private hospitals than in training and social security hospitals.^13^ Likewise, the study conducted in Malta in 1998 showed that the expectations of private hospital patients for service were higher than those of public hospital patients.^1^

Several patient characteristics have been associated with patient satisfaction including demographic factors, socio-economic status and general health status.^14, 15, 16^ Satisfaction is also influenced by the manner with which health care is delivered. The type of health care setting and characteristics of the medical provider, such as experience, age and gender, are related to patient satisfaction.^17, 18, 19^

Some medical-care satisfaction studies showed that people with poor health status had stronger feelings in either direction and that the most satisfied groups were those with good health or those suffering from chronic diseases.^4, 20, 21^ But other studies showed that the patient health status was not an important factor of satisfaction.^13^

A study conducted in Kerman hospitals, a city in south-eastern Iran, showed that the effect of age on satisfaction was not significant. But a study on the experience and satisfaction of patients with health care in 2002, pointed out that age is an important factor in reported satisfaction as in the case of the findings in six regions of Ethiopia.^4, 13, 22, 23^ Ethnic origin has also been found to have a relevance to patient satisfaction.^24^

Moreover, the Kerman hospital study cited above showed that patient satisfaction and the sex of the patient have a significant relationship; a similar finding was observed in the Wangmamyen Community Hospital study.^4, 25^ But a study conducted in six regions of Ethiopia showed that the sex of the patient is not a significant determinant of patient satisfaction in agreement with findings of a review of issues and concepts in 1997.^23, 26^

The level of education and satisfaction were found to have an inverse correlation. For instance, in the study of the six regions of Ethiopia mentioned above, educational status and marital status were observed to be significant determinants of the mean score for patient satisfaction.^4, 23^

The study carried out in 1998 on determinants of customer satisfaction with hospitals, showed that perceived competence of the hospital staff and their demeanour had the greatest impact on customer satisfaction. The quality of communication and the general condition of the facilities were also significant but less important in explaining customer satisfaction with hospital services.^27^ Besides, a clean and organised appearance of a hospital, its staff, its premises, restrooms, equipment, wards and beds can influence patients’ impressions about the hospital.^28^

Perceived waiting time is a strong predictor of patient satisfaction. If waiting time is longer than what is expected or considered inappropriate, dissatisfaction will arise no matter how long the actual waiting time.^29^ Moreover, many studies have shown that unfulfilled expectations are related to lower patient satisfaction.^25, 30, 31, 32, 33^ However, a study that focused on unmet expectations, reported that there was little support for the relationship between fulfilment of specific expectations and patient satisfaction.^24^ Nevertheless, studies indicated that patients have a tendency to infer the level of technical quality based on non-technical aspects.^34^

Throughout Ethiopia, there has been a significant increment in the number of private health care facilities in large urban centres, though the majority of the population depends on government hospitals for their health care.^35^ Addis Ababa, the capital city of Ethiopia, has 442 private clinics of various categories, 150 pharmacies (135 private, 3 NGO and 15 public), 83 private drug stores, 28 health centres, 7 nucleus health centres and health stations. Under the jurisdiction of the Addis Ababa City Administration Health Bureau (AACAHB) there are 5 public and 28 private hospitals with a total of 927 beds.^36^ By the year 2007–2008 total outpatient visits were 1 377 850 (the OPD attendance per capita was 0.60) and total inpatient admissions were 16 204 (admission rate was 5.1).^36^ However, there is no adequate documentation of the comparison of the level and determinants of patient satisfaction with the health services provided at public and private hospitals.

#### Significance of the study

Thus, this study aims to determine the level and determinants of patient satisfaction with the outpatient health service provided at public and private hospitals in Addis Ababa, central Ethiopia with the research question of: ‘Is there any difference in the level and determinants of patient satisfaction at public and private hospitals in Addis Ababa?’

## Ethical consideration

Before the start of the data collection, ethical clearance was secured from Jimma University, College of Public Health & Medical Sciences and the Addis Ababa Health Bureau. Permission was obtained from respective hospitals. Participation in the study was voluntary and based on the ability of each patient and/or caregiver to give verbal informed consent. Participants were guaranteed confidentiality of the information they gave and had the right to refuse participation or quit participation at any time during their involvement in the study.

## Trustworthiness

As it is detailed in the methods section, the study design and methods used to obtain data in this study are scientifically sound. Since the purpose of the study was to compare, a comparative cross-sectional study design was employed. The sampling procedure used to select the hospitals was random selection proceeded by systematic random sampling of the study subjects. Factor analysis was used since it enables proper treatment of the continuous data generated using the different scales. Finally, regression models were fitted to identify the independent predictors of patient satisfaction.

The scales used in this study have been used in other studies and pre-tests of the contextually adopted tools were carried out before the actual data collection. Multiple items were used to establish appropriate measurement properties of the selected constructs. At the end of each day, supervisors of data collection checked for the appropriate recording of all entries by the data collectors by randomly taking completed questionnaires. Methods used to measure concepts at the analysis stage are also well described above.

## Methods

### Participants

All patients who visited the OPDs at private and public hospitals in Addis Ababa during the study period were the source population for this study. The study population included patients who were identified by systematic random sampling at the OPDs of selected hospitals. During the interviews critically ill patients were excluded unless they had a caretaker who was willing to respond; parents or caregivers of children were interviewed at the paediatric OPDs.

By two population proportion formulae, taking Z a/_2_ = 1.96, Z_β_ = 0.84, p_1_ = 0.4 and p_2_ = 0.6 the calculated sample size for n_1_ was 152 and for both private and public hospitals it was 304 (2 * 152). Considering a 10% non–response rate and a design effect of 2, the total sample size was 668. In the calculation, p_1_ and p_2_ are patient satisfaction with OPD health services in Tigray, Ethiopia and Malta, respectively.^1, 37^ The proportion for private hospitals was taken from a study outside the country because we did not find similar studies in Ethiopia.

After identifying all private and public hospitals, all 5 public hospitals and 5 private general hospitals selected through the lottery method were included in the study. The number of respondents from each hospital was determined based on the proportion of patients who visited the OPDs during one month prior to the start of the study. The interval of the respondents for the study in private hospitals was determined by dividing the total number of patients in the private hospitals during the last one month, by the sample size for private hospitals. The same technique was applied at public hospitals.

### Design and setting

A comparative cross-sectional study was conducted from March 27 to April 30, 2010 in Addis Ababa.

### Procedures

The data were collected using a structured questionnaire. The questionnaire was developed in English and translated into Amharic (the local language) and retranslated back into English to ensure its consistency. The participants were interviewed by trained data collectors in the hospital, after having used the service and immediately before their exit. Consultation duration was recorded by the observation of the time patients spent in the examination room, from entry to exit.

### Analysis

Data was cleaned, edited, coded after it was entered into Epi Info version 3.4.3 and exported to SPSS version 16. Using SPSS version 16, descriptive statistics were used to determine indices. Factor analysis was done to identify factors that explained most of the variance observed in the population with regard to each scale. An analysis of variance for comparing responses from public and private hospital respondents, and multiple linear regression for identifying determinants of outpatient satisfaction at public and private hospitals, were done. A significance level of 0.05 was used in all cases.

Factor analysis was employed for all Likert scale instruments to extract factor(s) representing each of the scales and have factor scores, which facilitate treatment of the variables as continuous during further analysis. During all factor analysis procedures, principal axis factoring with eigenvalue greater than or equal to one extraction and varimax rotation methods were employed. The factors extracted for each of the scales, which had Cronbach's alpha value greater than 0.7, were used in a subsequent analysis. Whenever the scales had more than one factor extracted the factors were renamed according to the items contained in the factor extracted.

## Results

### Characteristics of the respondents

The respondents of the study were 626 patients or clients (313 from each group of the hospitals) with a response rate of 93.71%. At the public hospitals, the median age of the respondents was 30 years with a range of 15–75, whilst it was 32 years with range of 14–75 at the private hospitals. In both categories of hospitals, most of the respondents were females (61% at public and 53% at private). With regard to their religion, 61.7% and 73.2% of the respondents were orthodox Christians at the private and public hospitals, respectively. Even though the respondents in the two categories were quite homogeneous in their marital status (x^2^ = 4.13, df = 3, *p* = 0.248) and ethnicity (x^2^ = 7.212, df = 6, *p* = 0.302), they were significantly dissimilar in their occupational status (x^2^ = 43.642, df = 7, *p* = 0.005), educational status (x^2^ = 36.709, df = 5, *p* = 0.005) and wealth score distribution (F [624; 625] = 20.437, *p* < 0.0001) (Table 1).

**TABLE 1:** Socio-demographic characteristics of respondents at private and public hospitals in Addis Ababa, Central Ethiopia, April 2010.

Socio demographic variables	Public hospitals†		Private hospitals‡	
	
	*f*	%	*f*	%
**Sex**				
Male	122	38.98	147	47.0
Female	191	61.02	166	53.0
**Religion**				
Orthodox	229	73.2	193	61.7
Muslim	46	14.7	61	19.5
Protestant	35	11.2	49	15.7
Catholic	1	0.3	1	0.3
Others	2	0.6	9	2.9
**Educational status**				
Unable to read and write	41	13.1	21	6.7
Only read and write	5	1.6	7	2.20
1–4	19	6.1	8	2.6
5–8	59	18.8	34	10.9
9–12	113	36.1	119	38.0
Certificate and diploma	55	17.6	59	18.8
Degree and above	22	7.0	65	20.8
**Marital status**				
Single	120	38.3	115	36.7
Married	176	56.2	185	59.2
Divorced	8	2.6	2	0.6
Widowed	9	2.9	11	3.5
**Ethnicity**				
Amhara	149	47.6	133	42.5
Oromo	75	24.0	64	20.4
Gurage	25	8.0	28	8.9
Tigre	45	14.3	55	17.6
Others	18	6.1	33	10.6
**Occupation**				
Government employee	55	17.6	71	22.7
Merchant	38	12.1	69	22.0
NGO employee	37	11.8	68	21.7
House wife	72	23.0	50	16.0
Daily labourer	14	4.50	5	1.6
Student	51	16.30	28	8.9
Farmer	13	4.20	7	2.2
Others	33	10.5	15	4.8
**Wealth quintiles**				
Poorest	90	28.75	35	11.18
Second	69	22.00	56	17.89
Middle	57	18.20	69	22.05
Fourth	52	16.60	73	23.32
Richest	45	14.37	80	25.56

*f* = Frequency.

† *n* = 313.

‡ *n* = 313.

Most (90%) of the respondents at the private hospitals claimed that the reason for their visit was illness, whereas at the public hospitals, illness was the reason only for 63% the respondents (x^2^ = 64.23 df = 1, *p* = 0.005). Repeated visits were relatively higher at public hospitals (x^2^ = 0.243, df = 1, *p* = 0.622). Looked at specifically, a repeated visit of two times in the last month prior to the study was relatively higher at the public hospitals. Similarly, private hospital service users who visited the hospitals once during the last one month prior to the study were greater than those at the public hospitals (x^2^ = 18.96, df = 5, *p* = 0.002).

History of admission was relatively higher amongst public hospital service users. On the other hand, self-judged health status of private and public hospital service users was not significantly different (x^2^ = 5.69, df = 3 *p* = 0.128). Public hospital respondents reported that they received recommendation from others about the current hospital services more than private hospital patients (x^2^ = 6.97, df = 1, *p* = 0.008). However, private hospitals service users had higher expectation than public hospital service users about the service they should receive in their respective hospitals (x^2^ = 22.574, df = 3, *p* < 0.000).

#### Comparison of patient satisfaction and service characteristics

There is significant difference between the mean overall patient satisfaction scores at the private and public hospitals, (F [624; 625] = 26.229, *p* < 0.000). Moreover, there is also significant difference between the mean consultation duration, mean perceived providers technical competency score, mean perceived lack of provider's experience and ability score, mean perceived welcoming approach score, mean perceived body signalling score, mean perceived concern score, mean perceived empathy score, mean perceived encouragement score, mean interview day waiting time for hospitals services at the private and public hospitals.

#### Satisfaction with health care provided

The mean (±s.d.) waiting time for scheduled appointments at the public hospitals was 153.72 (±128.22) minutes with a range of 10–720. For private hospitals it was 87.15 (±88.89) minutes with a range of between 2–480. On the day of interview, the mean (±s.d.) waiting time at the public hospitals was 134.1 (±121.58) minutes with a range of between 5–738, whereas the mean (±s.d.) waiting time at the private hospitals was 80.1 (±79.9) minutes with a range of between 2–480.

The recorded consultation duration was 7.82 (±4.78) minutes at the public hospitals with a range of 1–45 minutes. But it was 10.59 (±6.01) minutes at the private hospitals with a range of 2–45 minutes. Of the respondents at the private hospitals, 65% reported that the consultation duration was enough whilst only 46% of the respondents described the same way at the public hospitals.

Concerning the overall satisfaction with the health care provided at the public hospitals, 18.2% of the patients were very satisfied whilst 47.9% the patients were just satisfied. At the private hospitals the corresponding proportions were 26.5% and 49.2%, respectively.

#### Predictors of patient satisfaction

At the private hospitals, out of all the socio-demographic variables only occupational and educational status were found to have a statistically significant association with the patient satisfaction score. Accordingly, patients who were students and housewives had 0.608 and 0.616 unit lower satisfaction scores respectively, as compared to patients who were government employees (95% CI, -1.015 to -0.202 and -0.998 to -0.233). The age of the respondents was excluded from this model because of multicollinearity with marital status. However, at the public hospitals none of the socio-demographic variables showed association with the patient satisfaction score (Table 2).

**TABLE 2:** Socio-demographic predictors of patient satisfaction at private hospitals in Addis Ababa, Central Ethiopia, April 2010.

Variables	*f*	%	Unstandardised Coefficients		Standardised Coefficients	Sig.	95% CI for B	
		
			B	Std. Error	Beta		Lower Bound	Upper Bound
**Sex**								
Male†	147	47	-	-	-		-	-
Female	166	53	.099	.108	.059	.360	-.114	.313
**Educational status**								
Unable to read & write	21	6.7	.159	.233	.047	.495	-.299	.616
Only read & write	7	2.2	.091	.354	.015	.797	-.605	.787
1–4	8	2.6	-.025	.316	-.005	.937	-.646	.596
5–8	34	10.9	-.122	.174	-.045	.486	-.465	.222
9–12†	119	38.0	-	-	-	-	-	-
Certificate & diploma	59	18.8	-.266	.141	-.124	.061	-.544	.012
Degree and above	65	20.8	-.336	.139	-.162	.017	-.610	-.062
**Religion**								
Orthodox†	193	61.7	-	-	-	-	-	-
Muslim	61	19.5	-.091	.140	-.043	.514	-.366	.184
Protestant	49	15.7	-.011	.147	-.005	.941	-.300	.278
Catholic	1	0.3	.596	.874	.040	.495	-1.123	2.315
Others	9	2.9	-.125	.292	-.025	.670	-.700	.451
**Ethnicity**								
Amhara†	133	42.5	-	-	-	-	-	-
Oromo	64	20.4	-.053	.132	-.026	.687	-.314	.207
Tigre	28	8.9	.022	.138	.010	.874	-.249	.293
Gurage	55	17.6	.044	.175	.015	.802	-.300	.388
Others	33	10.6	-.326	.511	-.038	.525	-1.332	.681
**Marital status**								
Single	115	36.7	-.109	.115	-.063	.343	-.335	.117
Married†	185	59.2	-	-	-	-	-	-
Divorced	2	0.6	.416	.609	.039	.495	-.782	1.615
Widowed	11	3.5	.304	.270	.067	.261	-.227	.835
**Occupation**								
Government Employee†	71	22.7	-	-	-	-	-	-
Merchant	69	22.0	-.619	.159	-.305	.000	-.931	-.306
NGO employee	68	21.7	-.183	.147	-.090	.214	-.472	.106
Housewife	50	16.0	-.616	.194	-.268	.002	-.998	-.233
Daily labourer	5	1.6	-.907	.434	-.135	.037	-1.761	-.053
Student	28	8.9	-.608	.207	-.207	.003	-1.015	-.202
Farmer	7	2.2	-.876	.371	-.154	.019	-1.606	-.146
Others†	15	4.8	-.784	.264	-.199	.003	-1.304	-.265

† = Reference category.

CI, Confidence Interval; *f*, Frequency.

On the other hand, the perceived cleanliness score of hospitals was found to be associated with the satisfaction score (Table 3). A standard deviation raise in the perceived cleanliness score resulted in 0.290 (*p* < 0.000) and 0.304 (*p* < 0.000) standard deviations increment in the satisfaction score at public and private hospitals, respectively.

**TABLE 3:** Perceived physical structure cleanliness, service cost and respondent wealth score as predictors of patient satisfaction at public and private hospitals in Addis Ababa, Central Ethiopia, April 2010.

Hospital types	Unstandardised Coefficients		Standardised Coefficients	95% CI for B	
		
	B	Std. Error	Beta	Lower Bound	Upper Bound
**Public**	-	-	-	-	-
Perceived service cost score	-.079	.059	-.073	-.194	.037
Perceived cleanliness of hospitals	.309	.058	.290	.194	.423
Wealth score	-.038	.051	-.041	-.139	.062
**Private**	-	-	-	-	-
Perceived service cost score	-.071	.050	-.078	-.169	.027
Perceived cleanliness of hospitals	.274	.049	.304	.179	.370
Wealth score	-.043	.052	-.045	-.146	.060

CI, Confidence Interval; *f*, Frequency.

The reasons for visit and interview day waiting time were associated with patient satisfaction at the public hospitals whilst at the private hospitals, recorded consultation duration and type of visit were found to have association with patient satisfaction. As shown in the model (Table 4), a standard deviation additional minute on recorded consultation duration could raise the satisfaction score by 0.197 (*p* < 0.000) standard deviations at private hospitals. On the other hand, patients who came repeatedly to private hospitals had 0.306 (95% CI, 0.116 to 0.495) unit higher satisfaction score than newcomers at the hospitals.

**TABLE 4:** Interview day service associated experiences as predictors of patient satisfaction at public and private hospitals in Addis Ababa, Central Ethiopia, April 2010.

Hospital type	Variables	*f*	%	Unstandardised Coefficients		Standardised Coefficients	95% CI for B	
		
				B	Std. Error	Beta	Lower Bound	Upper Bound
**Public**	Type of visit							
	New†	118	37.7	-	-	-	-	-
	Repeated	195	62.3	-.004	.120	-.002	-.241	.232
	Reason for visit Check-up							
	Illness†	197	62.9	-	-	-	-	-
	Check-up	116	37.1	.255	.120	.122	.019	.492
	Interview day waitingtime	-	-	-.001	.000	-.122	-.002	.000
	Transportation cost	-	-	.001	.001	.031	-.002	.003
	Recorded consultation duration	-	-	.000	.012	.000	-.023	.024
**Private**								
	Type of visit							
	New†	124	39.6	-	-	-	-	-
	Repeated	189	60.4	.306	.096	.178	.116	.495
	Reason for visit							
	Illness†	282	90.1	-	-	-	-	-
	Check-up	31	9.9	-.026	.155	-.009	-.331	.280
	Interview day waiting time	-	-	.000	.001	-.080	-.002	.000
	Transportation cost	-	-	.000	.001	-.043	-.003	.001
	Recorded consultation duration	-	-	.028	.008	.197	.012	.043

† Reference category.

CI, Confidence Interval; *f*, Frequency.

On the previous service related experiences model for both categories of hospitals, the variables that showed significant association with patient satisfaction scores were self-judged health status and expectation about hospital services (Table 5). Public hospital patients who judged their health status as 'getting worse’ had 0.657 (95% CI, -0.992 to -0.323) unit lower satisfaction score than patients who judged their current health status as 'well’. Besides, having low expectation for hospital services lowered the satisfaction score of public and private hospital service users by 0.869 (95% CI, -1.185 to -0.553) and 1.386 (95% CI, -1.784 to -0.988) unit respectively, compared to their counterpart respondents of high expectation.

**TABLE 5:** Previous patient experiences as predictors of patient satisfaction at public and private hospitals in Addis Ababa, Central Ethiopia, April 2010.

Hospital type	Variables	Frequency	%	Unstandardised Coefficients		Standardised Coefficients	95% CI Interval for B	
		
				B	Std. Error	Beta	Lower Bound	Upper Bound
**Public**	Waiting time for scheduled appointment	-	-	0	0	0.045	0	0
	History of admission	-	-	0.034	0.13	0.014	-0.221	0.289
	Recommendation from others	-	-	-0.057	0.116	-0.025	-0.284	0.171
	Frequency of visit (last one month)	-	-	-	-	-	-	-
	Once	34	10.9	-0.074	0.175	-0.023	-0.419	0.271
	Twice†	50	16	-	-	-	-	-
	3–4 times	38	12.1	-0.159	0.168	-0.051	-0.488	0.171
	> 5 times	13	4.2	-0.051	0.26	-0.01	-0.563	0.462
		-	-	-	-	-	-	-
	Self-judged health status	-	-	-	-	-	-	-
	Very well	38	12.1	0.433	0.164	0.14	0.109	0.756
	Well†	158	50.48	-	-	-	-	-
	No change	81	25.9	-0.746	0.127	-0.324	-0.995	-0.496
	Getting worse	36	11.5	-0.657	0.17	-0.208	-0.992	-0.323
	Expectation about the service	-	-	-	-	-	-	-
	High†	114	36.42	-	-	-	-	-
	Medium	126	40.3	-0.281	0.119	-0.137	-0.515	-0.047
	Low	45	14.4	-0.869	0.16	-0.302	-1.185	-0.553
	None	28	8.9	-0.179	0.194	-0.051	-0.561	0.202
**Private**	Waiting time for scheduled appointment	-	-	0	0	-0.099	0	0
	History of admission	-	-	-0.148	0.126	-0.06	-0.396	0.099
	Recommendation from others	-	-	-0.158	0.089	-0.091	-0.333	0.017
	Frequency of visit (last one month)	-	-	-	-	-	-	-
	Once	-	-	-	-	-	-	-
	Twice†	53	16.9	-0.024	0.124	-0.011	-0.268	0.22
	3–4 times	21	6.7	-	-	-	-	-
	>5 times	28	8.9	0.112	0.158	0.038	-0.199	0.422
		17	5.4	0.044	0.192	0.012	-0.333	0.421
	Self-judged health status	-	-	-	-	-	-	-
	Very well	45	14.4	0.273	0.124	0.114	0.03	0.517
	Well†	168	53.67	-	-	-	-	-
	No change	57	18.2	-0.399	0.115	-0.183	-0.625	-0.172
	Getting worsen	43	13.7	-0.282	0.128	-0.116	-0.535	-0.03
	Expectation about the service	-	-	-	-	-	-	-
	High†	154	49.2	-	-	-	-	-
	Medium	120	38.3	-0.445	0.091	-0.257	-0.625	-0.265
	low	14	4.5	-1.386	0.202	-0.341	-1.784	-0.988
	None	25	8.0	-0.722	0.16	-0.233	-1.038	-0.406

† Reference category.

CI, Confidence Interval; *f*, Frequency.

In the fifth model, perceived adequacy of consultation duration; perceived empathy; perceived technical competency; perceived lack of experience and ability; and perceived welcoming approach, appeared as predictors of patient satisfaction at both categories of hospitals. In addition, perceived body signalling was predictor at public hospitals. More specifically, a standard deviation increment in perceived technical competency score added an estimated 0.269 (*p* < 0.001) and 0.486 (*p* < 0.001) standard deviations to the patient satisfaction score at public and private hospitals, respectively. On the contrary, one standard deviation perceived lack of experience and ability, lowered the satisfaction score at public hospitals by 0.094 (*p* = 0.036) standard deviations and in private hospitals by 0.125 (*p* = 0.003) standard deviations (Table 6).

**TABLE 6:** Perceived service characteristics as predictors of patient satisfaction at public and private hospitals, Addis Ababa, Central Ethiopia, April 2010.

Hospital type	Variables	Unstandardised Coefficients		Standardised Coefficients	95% CI for B	
		
		B	s.d. Error	Beta	Lower Bound	Upper Bound
**Public**	Perceived adequacy of consultation duration	.131	.041	.151	.050	.211
	Perceived concern	.052	.057	.046	-.060	.163
	Perceived empathy	.127	.056	.110	.016	.238
	Perceived encouragement	.101	.065	.078	-.026	.229
	Perceived providers technical competency	.287	.057	.269	.176	.399
	Perceived lack of experience and ability	-.104	.049	-.094	-.200	-.007
	Perceived welcoming approach	.240	.063	.216	.116	.365
	Perceived body signalling	.129	.056	.105	.020	.239
**Private**	Perceived adequacy of consultation duration	.191	.042	.200	.109	.273
	Perceived concern	.042	.040	.045	-.036	.119
	Perceived empathy	.121	.045	.128	.033	.210
	Perceived encouragement	-.024	.045	-.022	-.113	.065
	Perceived welcoming approach	.099	.043	.109	.014	.183
	Perceived body signalling	.017	.041	.016	-.064	.099
	Perceived open posture	-.039	.047	-.035	-.132	.053
	Perceived technical competency	.447	.041	.486	.366	.528
	Perceived lack of experience and ability	-.118	.040	-.125	-.197	-.039

CI, Confidence Interval.

All significant predictors of patient satisfaction in the above models were entered into a final regression model and the final predictors of the satisfaction score for patients at private and public hospitals were identified and shown in Tables 7 and 8. Accordingly, patients who reported adequate consultation duration had 0.095 (95% CI, 0.020 to 0.171) and 0.011 (95% CI, 0.000 to 0.021) unit higher satisfaction scores than those who reported that consultation duration was not adequate at public and private hospitals, respectively. Besides, at private hospitals a standard deviation additional minute on recorded consultation duration raised the patient satisfaction score by 0.077 (*p* = 0.043) standard deviations. At private hospitals having low expectation for hospitals services decreased patient satisfaction by an average of 0.929 (95% CI, -1.237 to -0.621) unit and at public hospitals by an average 0.521 (95% CI, -0.778 to -0.264) unit as compared to patients who had high expectations. An increase of one standard deviation in perceived providers’ technical competency score causes 0.237 (*p* < 0.0001) and 0.409 (*p* < 0.0001) a standard deviations rise in the satisfaction score at public and private hospitals, respectively.

**TABLE 7:** Final predictors of patient satisfaction at public hospitals in Addis Ababa, Central Ethiopia, April 2010.

Variables	*f*	%	Unstandardised Coefficients		Standardised Coefficients	95% Confidence Interval for B	
		
			B	Std. Error	Beta	Lower Bound	Upper Bound
Reason for visit	-	-	-	-	-	-	-
Illness†	197	62.9	0.055	0.085	0.026	-0.113	0.223
Check-up	116	37.1	-	-	-	-	-
Interview day waiting time	-	-	0	0	-0.074	-0.001	0
Self-judged health status	-	-	-	-	-	-	-
Very well	38	12.1	0.142	0.134	0.046	-0.121	0.405
Well†	158	50.48	-	-	-	-	-
No change	81	25.9	-0.431	0.101	-0.187	-0.631	-0.232
Getting worse	36	11.5	-0.436	0.137	-0.138	-0.705	-0.167
Expectation about the service	-	-	-	-	-	-	-
High†	114	36.42	-	-	-	-	-
Medium	126	40.3	-0.146	0.093	-0.071	-0.329	0.037
Low	45	14.4	-0.521	0.131	-0.181	-0.778	-0.264
None	28	8.9	-0.076	0.152	-0.021	-0.375	0.223
Perceived adequacy of consultation duration	-	-	0.095	0.038	0.11	0.02	0.171
Perceived empathy	-	-	0.067	0.052	0.058	-0.035	0.169
Perceived technical competency	-	-	0.253	0.05	0.237	0.155	0.351
Perceived lack of experience and ability	-	-	-0.076	0.045	-0.069	-0.164	0.013
Perceived welcoming approach	-		0.211	0.054	0.19	0.104	0.318
Perceived body signalling	-	-	0.149	0.051	0.122	0.05	0.249
Perceived cleanliness of hospitals	-	-	0.185	0.044	0.174	0.099	0.272

† Reference category.

CI, Confidence Interval; *f*, Frequency.

**TABLE 8:** Final predictors of patient satisfaction at private hospitals in Addis Ababa, Central Ethiopia April 2010.

Variables	*f*	%	Unstandardised Coefficients		Standardised Coefficients	95% CI for B	
		
			B	Std. Error	Beta	Lower Bound	Upper Bound
**Educational status**							
Unable to read & write	21	6.7	-0.021	0.15	-0.006	-0.316	0.273
Only read and write	7	2.2	-0.011	0.229	-0.002	-0.461	0.439
Grade 1–4	8	2.6	0.174	0.206	0.033	-0.233	0.58
Grade 5–8	34	10.9	-0.128	0.11	-0.047	-0.345	0.089
Grade 9–12†	119	38	-	-	-	-	-
Certificate & diploma	59	18.8	-0.191	0.092	-0.089	-0.372	-0.01
Degree and above	65	20.8	-0.221	0.091	-0.107	-0.401	-0.041
**Occupation**							
Government employee†	71	22.7	-	-	-	-	-
Merchant	69	22	-0.217	0.099	-0.107	-0.412	-0.021
NGO employee	68	21.7	0.007	0.092	0.004	-0.175	0.189
Housewife	50	16	-0.312	0.121	-0.136	-0.55	-0.074
Daily labourer	5	1.6	-0.231	0.277	-0.034	-0.776	0.314
Student	28	8.9	-0.236	0.125	-0.08	-0.483	0.011
Farmer	7	2.2	-0.333	0.243	-0.059	-0.811	0.145
Others	15	4.8	-0.393	0.161	-0.1	-0.711	-0.076
**Type of visit**							
New†	124	-	-	-	-	-	-
Repeat	189	60.4	0.103	0.068	0.06	-0.031	0.238
Recorded consultation duration	-	-	0.011	0.005	0.077	0	0.021
**Self-judged health status**							
Very well	45	14.4	0.041	0.093	0.017	-0.142	0.224
Well†	168	53.67	-	-	-	-	-
No change	57	18.2	-0.208	0.09	-0.096	-0.386	-0.031
Getting worsen	43	13.7	-0.161	0.099	-0.066	-0.356	0.034
**Expectation about the service**							
High†	154	49.2	-	-	-	-	-
Medium	120	38.3	-0.047	0.074	-0.027	-0.193	0.098
Low	14	4.5	-0.929	0.157	-0.228	-1.237	-0.621
None	25	8.0	-0.311	0.127	-0.1	-0.561	-0.062
Perceived adequacy of consultation duration	-	-	0.142	0.039	0.149	0.065	0.219
Perceived empathy	-	-	0.097	0.042	0.102	0.014	0.18
Perceived welcoming approach	-	-	0.102	0.041	0.113	0.021	0.183
Perceived technical competency	-	-	0.376	0.04	0.409	0.297	0.455
Perceived lack of experience and ability	-	-	-0.075	0.037	-0.079	-0.148	0
Perceived cleanliness of hospitals	-	-	0.05	0.036	0.056	-0.02	0.121

† Reference category.

CI, Confidence Interval; *f*, Frequency.

Whilst none of the socio-demographic variables were picked as final predictors of patient satisfaction at public hospitals, occupational and educational status were picked for private hospitals. Patients who were housewives and merchants had 0.312 (95% CI,-0.550 to -0.074) and 0.217(95% CI, -0.412 to -0.021) unit lower satisfaction scores, respectively, as compared to patients who were government employees.

## Discussion

Health services that are provided in health care institutions need to be satisfactory so as to provide the intended effects of the services. This study has clearly demonstrated that the proportion of patients who were at least satisfied is higher at private hospitals. The difference was also found to be statistically significant, F (624; 625) = 26.229, *p* < 0.005. This finding is quite comparable with other studies which were conducted in different parts of the country and elsewhere in developing countries.^4, 10, 11, 12^ For example, a study done in a community hospital in Thailand depicted that the overall satisfaction of patients of health services was high, medium and low for 23.3%, 61.4% and 15.3% of the respondents, respectively.^25^

Attempts made to identify determinants of patient satisfaction, revealed that common determinants of patient satisfaction at private and public hospitals were self-judged health status, expectation about the services, perceived health care providers technical competency, perceived welcoming approach and perceived adequacy of consultation duration. But perceived body signalling and perceived cleanliness of the hospitals were determinants only for public hospital patients. On the other hand, perceived health care providers’ empathy, perceived lack of experience and ability, recorded consultation duration, educational status and occupation were unique determinants of patient satisfaction at private hospitals. Most of these variables were also found to be determinants of patient satisfaction in studies carried out elsewhere.^20, 21, 24, 26, 28, 29, 31^

Previous studies presented controversial findings regarding the relationship between a self-judged health status and patient satisfaction. Some reported that it had a positive association with satisfaction, but others claimed otherwise.^13, 20^ Still others reported that people with poor health had stronger feelings in either direction and that the most satisfied groups were those with good health or those suffering from chronic diseases.^4, 22^ In this study, however, it was found that a self-judged health status has positive association with patient satisfaction.

Expectation about hospital service may be influenced by previous experience of the service or based on information obtained from others. Patients at public hospitals had lower expectations about the services to be received than those at private hospitals (x^2^ = 22.574, df = 3, *p* ≤ 0.005). This finding is consistent with the finding in Malta.^1^ On the other hand, it is worth noting that this variable was an important determinant factor for satisfaction at both categories of hospitals of the present study and other studies carried out elsewhere previously.^24, 26, 32, 33, 34^

It is obvious that having good communication improves the outcome of the patient-physician interaction. Non-verbal communication is part of the interaction in service provision activities and can easily be misinterpreted, and in effect has an impact on patient satisfaction. In this study, both of the non-verbal communication factors – perceived welcoming approach and perceived body signalling – were significant determinants of patient satisfaction at public hospitals and the only perceived welcoming approach at private hospitals, which is quite similar with other findings though they reported it in general form as 'nonverbal communication.'^20, 28, 31^

Obviously, patients come to health care institutions with different health problems and they seek remedy for their problems. This puts them in need to be well heard whilst they are talking about their problems. To have an adequate consultation duration may allow health care providers to do so and know about their client and their health problems for consequent decision and effective consultation. In this study recorded consultation duration at private hospitals was 10.59 (±6.01) and at public hospitals was 7.82 (±4.78). Furthermore, 65% and 46% of the respondents of private and public hospitals respectively, reported that the consultation duration was long enough. On top of that, perceived adequacy of consultation duration was a determinant of patient satisfaction in both categories of hospitals whilst recorded consultation duration was so only at the private hospitals. This finding is consistent with that of Birhanu et al. in rural parts of central Ethiopia and implies the importance of sufficient consultation duration when providing satisfactory health services to clients in both settings.^19^ In order to make the services easy to get by the patients, health care providers may hurry to the next case without giving enough consultation time for the patients at hand. However, this fashion of addressing the problem of the service has its own impact on the receiver of the services and causes dissatisfaction. Moreover, hurry may undermine health care providers’ empathy, perceived technical competency and other important characteristics of the services.

Different studies indicated that patients have a tendency to infer the level of technical quality based on non-technical aspects, such as care providers’ compassion and empathy, responsiveness and service coordination amongst individual health care personnel.^22, 27^ In this study it was found that perceived technical competency (for both categories of hospitals) and perceived empathy (for private hospitals) had a positive association with patient satisfaction, this is similar with earlier findings. ^22, 28^ On the other hand, perceived lack of experience and ability had a negative association with patient satisfaction at private hospitals.

In conclusion, although patients at the private hospitals were more satisfied than patients at the public hospitals, the majority of the identified determinants of patient satisfaction in this study were service related and common to both settings. However, perceived body signalling and perceived cleanliness of hospitals were unique determinants at public hospitals. On the other hand, perceived empathy, perceived lack of experience and ability, recorded consultation duration, educational status and occupation were unique determinants of patient satisfaction at private hospitals.

### Practical implications

Despite the difference in the regulatory framework and other characteristics, both public and private hospitals may spend years on working towards health care quality. However, assessment of patient satisfaction has been widely recommended as a proxy indicator of the quality of health care in the literature. This is particularly relevant in settings where other forms of evidence base for service improvement are lacking.

In Ethiopia, the current health system allows mixed functioning of public and private health facilities, which have raised a concern about the quality of care being delivered in both public and private facilities. In Addis Ababa there are many private and public hospitals compared to other parts of the country. However, there is no adequate documentation of the comparison of the level and setting specific determinants of patient satisfaction with the health services provided at these hospitals. The findings of this study are of practical importance to both settings by enabling health managers to look into the major areas of concern, which could result in substantial improvements in the provision of patient or client-friendly services. The findings will also help the managers to compare the situation in their facility with those of others.

### Limitations of the study

Finally, it is worth noting that there is an inherent difference of purpose between the two categories of hospitals compared in this study. This may contribute to the differences in patient satisfaction levels and the determinants. Social desirability bias is also likely in this study as the respondents were interviewed in the compound of the health facility. Moreover, patients may experience a relatively short-lived 'halo effect’ whereby they feel more satisfied immediately after their consultation than they do afterwards. It should also be noted that the reliance on the response of parents or caregivers for their children might introduce surrogate bias. We would also like the reader to note that findings in this study are applicable to the study area with cautious generalisation to other similar settings, and don't have the nature of in-depth description which could have been achieved if qualitative methods were employed.

### Recommendations

It is recommended that hospitals in both categories should work to improve the ability of their employees, particularly health professionals, to win the interests of the clients and have a physical structure that better fits the expectations of the patients as indicated by the identified determinants of patient satisfaction in this study.

## Conclusion

Health managers in both settings should design in-service training to enable their health care providers to demonstrate better relational empathy, technical competency and non-verbal behaviours during consultations. It is also advisable to note the unique predictors of patient satisfaction in each setting whilst working toward patient-centeredness.
